# Cloning and Characterization of a New Site-Specific Methyl-Directed ElmI Endonuclease Recognizing and Cleaving C5-methylated DNA Sequence 5’-G(5mC)^NG(5mC)-3’

**Published:** 2016

**Authors:** V. A. Chernukhin, D. A. Gonchar, M. A. Abdurashitov, O. A. Belichenko, V. S. Dedkov, N. A. Mikhnenkova, E. N. Lomakovskaya, S. G. Udal’yeva, S. Kh. Degtyarev

**Affiliations:** SibEnzyme, Timakova St., 2/12, 630117, Novosibirsk, Russia

**Keywords:** methyl-directed site specific endonuclease, MD-endonuclease, epigenetics, methylome analysis, DNA endonuclease gene cloning

## Abstract

Putative open reading frames of MD-endonucleases have been identified in Enterobacteria genomes as a result of the search for amino acid sequences homologous to MD-endonuclease BisI. A highly conserved DNA primary structure of these open reading frames in different genera of Enterobacteria (*Escherichia*, *Klebsiella* and *Cronobacter*) has allowed researchers to create primers for PCR screening, which was carried out on Enterobacteria DNA collected from natural sources. The DNA fragment, about 440 bp in length, was amplified by use of the genomic DNA of a wild *E.coli *LM N17 strain as a template and was inserted into the pMTL22 vector. Endonuclease activity was detected in an *E.coli *ER 2267 strain transformed with the obtained construction. A new enzyme named ElmI was purified by chromatographic techniques from the recombinant strain biomass. It was discovered that similarly to BisI this enzyme specifically cleaves the methylated DNA sequence 5’-GCNGC- 3’ before the central nucleotide “N” if this sequence contains two 5-methylcytosines. However, unlike BisI, ElmI more efficiently cleaves this sequence if more than two cytosine residues are methylated.

## INTRODUCTION

Methyl-directed site specific endonucleases (MD-endonucleases) recognize and cleave DNA at specific methylated sequences, leaving unmethylated DNA untouched. In the last nine years, more than 10 prototypes of these enzymes have been described. They have different recognition sites, which are cleaved only when the cytosine residues within them are C5-methylated.

In contrast to restriction endonucleases, MD-endonucleases recognize not only a specific nucleotide sequence and relative hydrolysis position in this sequence, but also a specific pattern of methylation. Therefore, different MD-endonucleases, even those with similar recognition sites, may cleave DNA differently based on its methylation pattern. Enzymes that recognize the methylated 5’-GCNGC-3’ sequence are a great example. Among the enzymes that recognize this site and cleave DNA after the central nucleotide “N” BlsI [1] cleaves this sequence if it contains at least two and PkrI [2] if it contains at least three 5-methylcytosine residues. Among the enzymes that cleave the sequence before the central nucleotide “N,” MD-endonuclease BisI [3] cleaves the 5’-GCNGC-3’ sequence if it contains two 5-methylcytosine residues (and much less efficiently, if there is only one), whereas GluI [4] needs four 5-methylcytosine residues.

We have described a new representative of this group of enzymes, MD-endonuclease ElmI, that recognizes the methylated 5’-GCNGC-3’ sequence and cleaves it before the central nucleotide “N” (to form 5’-overhanging single-nucleotide ends) if the sequence contains at least two 5-methylcytosine residues; the enzyme activity increases by an order of magnitude if three or four cytosines in the recognition site are methylated.

## MATERIALS AND METHODS

**PCR screening of Enterobacteria DNA collected from natural sources and production of pElmI plasmid with the new MD-endonuclease gene**

Coliform bacteria were isolated from natural sources (sewage water) on a selective Endo medium according to [5]. Eight to twenty strains with different morphological characteristics were collected from each sample inoculation (LM, LT, LP, and LV series).

Chromosomal DNA was isolated from these strains and screened by PCR using the primers listed below. The fragment amplified using DNA from one of the wild-type strains was inserted into the pMTL22 plasmid [6] at the BglII and FauNDI restriction sites.

After transformation of the *E. coli *ER2267 cells, the clones carrying the target plasmid (called pElmI) were plated on Petri dishes with an agarized LB medium supplemented with ampicillin (50 μg/ml). The clones were grown overnight at 37°C, subcultured to separate dishes with ampicillin (100 μg/ml), and allowed to grow overnight for further analysis.

**Production of biomass of the recombinant ElmI producer strain and assay of the target activity**

The recombinant clone of the *E. coli *ER2267 strain, which was transformed with pElmI plasmid, was transferred from Petri dishes to a bottle with 200 ml of a LB broth supplemented with ampicillin (100 μg/ml) using an inoculation loop. The inoculum was grown overnight using a thermostatted shaker (37°C, 120 rpm).

5 ml of inoculum were seeded into 20 bottles with 200 ml LB broth supplemented with ampicillin (100 μg/ml) and 0.5 mM isopropyl-β-*D*thiogalactopyranoside (IPTG).

The culture was grown for 10 hours in a thermostatic shaker (120 rpm), and then an aliquot (1 ml) for the enzyme activity assay was withdrawn and transferred to a 1.5 ml Eppendorf tube. The cells were pelleted using a 5416 Eppendorf tabletop centrifuge (Eppendorf GmbH, Germany, 12,000 rpm, 2 min). The supernatant was removed, and the precipitate was resuspended in 0.2 ml of a lysis buffer (10 mM Tris-HCl, pH 8.5, 0.1 mg/ml lysozyme, 0.5 M NaCl, 1 mM EDTA, 0.1% Triton X-100).

The activity of the enzyme in the lysate was assayed in 20 μl of the reaction mixture, which contained pFsp4HI3 plasmid, pre-linearized with DriI restriction enzyme, as a DNA substrate [4]. The linearization was performed in a SE buffer “W” (10 mM Tris-HCl (pH 8.5 at 25°C), 10 mM MgCl_2_, 100 mM NaCl, 1 mM dithiothreitol) for 2 hours at 37°C. The amount of ElmI enzyme sufficient for complete hydrolysis of 1 μg of pFsp4HI3 DNA (2 hours, 37°C, SE buffer “W”) was taken as 1 unit activity of the enzyme. The presence or absence of hydrolysis of the DNA substrate was determined by electrophoresis in 1% agarose gel.

The cells of all of the produced biomass were pelleted using a J2-21 centrifuge (30 min, 8,000 rpm, JA- 10 rotor, Beckman, USA) and frozen.

**Production of ElmI enzyme preparation**

All procedures for isolation and purification of the enzyme preparation were performed at 4°C using the following solutions:

- Buffer A: 10 mM Tris-HCl, pH 7.5; 0.1 mM EDTA; 7 mM 2-mercaptoethanol;

- Buffer B: 10 mM K-phosphate buffer, pH 7.4; 0.1 mM EDTA; 7 mM 2-mercaptoethanol.

pFsp4HI3 in the SE buffer “W,” digested for 15 min in 20 μl of the reaction mixture by adding aliquots (1 μl) of chromatographic fractions, was used as the DNA substrate [4] to determine the activity of the enzyme.

*Isolation of the crude extract*. The biomass (8 g) was suspended in 30 ml of buffer A containing 0.2 M NaCl, 1 mM phenylmethylsulfonyl fluoride (PMSF), 0.1% Triton X-100; and 0.1 mg /mL lysozyme. The cells were disrupted by sonication on a Soniprep 150 sonicator (MSE, UK, 2 cm adapter, amplitude of 20 μm), using four 1-minute periods followed by 1-minute intervals to cool the suspension in an ice bath.

Cell debris was removed by centrifugation at 15,000 rpm for 30 min (JA-20 rotor, J2-21 centrifuge, Beckman, USA).

*Chromatography on phosphocellulose P-11. *The crude extract was diluted twofold with buffer A and applied to a column with phosphocellulose P-11 (30 ml), pre-equilibrated with buffer A containing 0.1 M NaCl, and washed with 160 mL of the same buffer. The adsorbed material was eluted using a linear gradient of NaCl (0.1 to 1 M) in buffer A (total volume of 800 ml); 30 fractions were collected, and fractions 16 to 22 (0.35 to 0.47 M NaCl) exhibiting target activity were pooled together.

*Chromatography on hydroxylapatite. *The pooled fractions were applied to a column with hydroxylapatite (2 ml), pre-equilibrated with buffer B containing 0.05 M NaCl, and washed with 10 ml of the same buffer. The adsorbed material was eluted using a linear gradient of K-phosphate buffer (pH 7.4) containing 0.05 M NaCl (0.01 to 0.1 M, total volume of 30 ml). A gradient of 20 fractions was collected, and fractions 8 to 12 (0.044 to 0.056 M K-phosphate) exhibiting ElmI activity were pooled together. The pooled fractions were dialyzed for 1 h against 300 ml of buffer A.

*Chromatography on heparin-agarose. *The dialyzed fractions were applied to a column with heparin-agarose (2 ml), pre-equilibrated with buffer B containing 0.05 M NaCl, and washed with 4 ml of the same buffer. The adsorbed material was eluted using a linear gradient of NaCl (0.05 to 1 M) in buffer B (a total volume of 30 ml); 20 fractions were collected, and fractions 12 and 13 (0.62 to 0.67 M NaCl) exhibiting the target activity were pooled together.

*Concentration, activity assays and storage. *The pooled fractions were dialyzed for 20 hours against a 15-fold volume of buffer B with 50% glycerol and 0.2 M NaCl, and stored at -20°C.

The activity was assayed by adding eight consecutive two-fold dilutions of the enzyme preparation (2, ^1^/_2_, . μl, etc.) to 20 μl of the reaction mixture containing 0.5 μg of pFsp4HI3/DriI DNA in the SE buffer “W,” followed by 2 h incubation at 37°C. The SE buffer for enzyme dilution “B100” (10 mM Tris-HCl (pH 7.6 at 25°C), 50 mM KCl, 0.1 mM EDTA, 200 μg/ml BSA, 1 mM dithiothreitol, 50% glycerol) was used to dilute enzyme preparations. The reaction was stopped by adding 1 μl of a stop buffer (50% glycerol, 10 mM EDTA, 0.2% bromophenol blue) to each reaction mixture.

Sanger sequencing was used to **determine the DNA sequence **on a ABI 3130xI Genetic Analyzer automatic sequencer (Applied Biosystems, USA) according to the manufacturer’s instructions.

**Preparations **of enzymes, DNA, deoxynucleoside triphosphates, and synthetic oligonucleotides, as well as the molecular weight markers (1 kb Ladder and Lambda/StyI) used in this work, were produced by SibEnzyme (Russia).

## RESULTS AND DISCUSSION

**Cloning of the new MD-endonuclease ElmI gene and comparative analysis of nucleotide and amino acid sequences**

Previously, we found MD-endonucleases in bacteria from different taxonomic groups, but mostly in representatives of the Microbacteriaceae and Bacillaceae families. The earlier screening of cell lysates did not allow us to identify similar site-specific enzymes in Enterobacteriaceae strains, which may indicate either the absence or the extremely low activity of MD-endonucleases in this group of bacteria. To resolve this issue, we decided to use bioinformatic rather than the biochemical method to search for homologous enterobacterial proteins.

The PSI-BLAST (https://blast.ncbi.nlm.nih.gov) software was used to screen the database of Enterobacteriaceae amino acid sequences for sequences homologous to the previously described MD-endonuclease BisI (GenBank AJW87312) [7]. Two search iterations revealed ~50 enterobacterial amino acid sequences which were homologous to the BisI sequence (32–48% similarity, 17–30% identity). The roles of all these homologous proteins have been unknown. The nucleotide sequences of the corresponding genes were extracted from the GenBank database and compared to each other. It has been shown that the sample contains two groups of highly homologous genes. The first group includes genes of four putative proteins with a length of 143– 144 amino acid residues from the bacteria of genera* Escherichia *(GenBank accession numbers ACT43858 and AKN48098), *Cronobacter *(CCJ93299), and *Klebsiella* (KEG36084). A comparison of these genes to each other revealed a 93–99% identity. The second group contained enterobacterial genes which encode proteins with a length of 290 amino acid residues, whose N-terminal portion is homologous to the BisI protein (GenBank protein accession numbers: KFC97828, WP_000794335, WP_000794336, WP_000794337, WP_001655794, WP_004952390, WP_008806407, WP_021557167, WP_025912430, WP_032653240, WP_032671961, and WP_033070923 ). The degree of nucleotide sequence identity in this group is 83–99%.

**Fig. 1:**
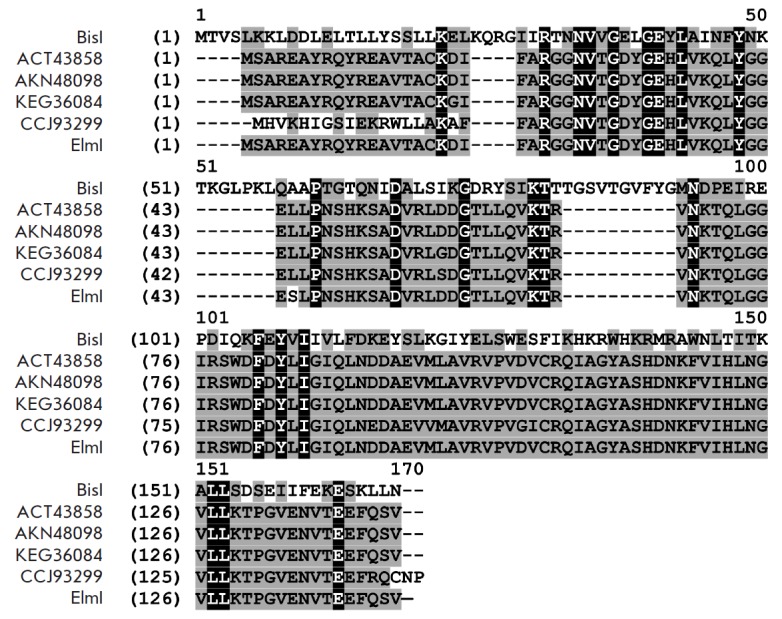
Alignment of the amino acid sequences of BisI and ElmI with the most homologous proteins from enterobacteria. The designations of amino acid sequences correspond to GenBank numbers, as described in the text. Amino acids that are identical in all presented protein sequences are shown in white on a black background. Amino acids with similar physical and chemical properties are shown in black on a gray background.

*Figure 1* shows multiple alignment of the amino acid sequences of the four highly homologous enterobacterial proteins from the first group, which have a BisI sequence. The sequence of endonuclease ElmI, which was detected by PCR screening, is also shown (see the description below).

**Fig. 2:**
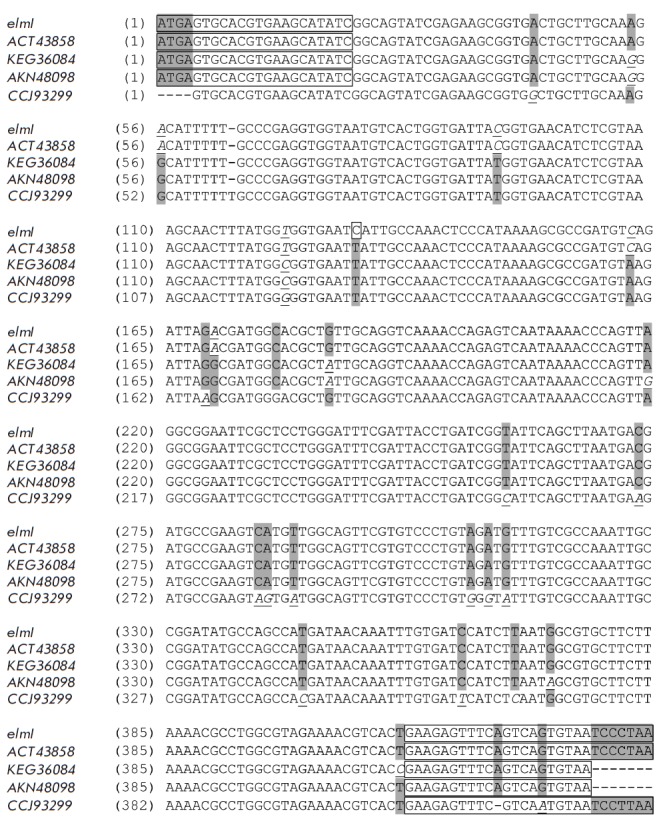
Alignment of the nucleotide sequence of the putative gene encoding ElmI (*elmI *gene) with the most homologous DNA sequences. Nucleotide sequences are identified by GenBank accession numbers for the corresponding encoded proteins. Nucleotides identical for most, but not for all, of the analyzed sequences of 5 genes are shown on a gray background. Nucleotides that are not found in the majority of the sequences are shown in italics and underlined. The single nucleotide by which the *elm*I gene differs from the nearest homologues from *Escherichia coli *BL21(DE3) and C41(DE3) is indicated by a frame. The sequences on the 5’ and 3’ ends corresponding to the primers by which the PCR screening for isolation of coliform bacteria genomic DNA from natural sources was performed are indicated by frames as well.

Multiple alignment of the corresponding enterobacterial genes (*Figure 2*) revealed that their nucleotide compositions also have a high degree of identity, even though the host organisms belong to different genera. The sequence of the *elmI *gene, established in this work, is added to the alignment.

The high degree of sequence identity for the genes from the two aforementioned groups, which holds true for their end sites, allowed us to select primers for PCR screening of wild-type strains for the presence of similar genes. To search for genes encoding proteins related to the first group of proteins, we used primers containing the recognition sites of FauNDI and BamHI restriction endonucleases for inserting PCR fragments into a plasmid vector:

Esp-1 5’-CCCCCATATGAGTGCACGTGAAGCATATC- 3’

Esp-2 5’-CGCGGATCCTTAGGGATTACACTGACTGAAACTCTTC- 3’.

For PCR screening for genes similar to the second group of genes, the following primers were synthesized:

Esp-3 – 5’-TTGAAAAATAATCATTTAACATCATATG- 3’

Esp-4 – 5’-TCACTCCAGAACGCTGATAAGTTT- 3’.

Genomic DNA was isolated from 64 strains of coliforms bacteria detected in sewage waters and used as a template for PCR. Amplification with the Esp-3 and Esp-4 primers (the second group of genes) did not result in a fragment of the expected length (~870 bp) in any of the matrix DNA. The use of the Esp-1 and Esp-2 primers (the first group of genes) resulted in a PCR fragment of the expected length (~430 bp) in one DNA sample.

This amplified fragment was treated with the Bam- HI and FauNDI restriction enzymes and ligated into the pMTL22 vector, which had been previously digested with FauNDI and BglII. The resulting plasmid, named pElmI, was used to transform the *E. coli *ER2267 cells.

Taxonomic specificity of the original strain whose genomic DNA was amplified to obtain the fragment was determined using conventional biochemical and morphological criteria [8], and by analyzing the structure of the 16S rRNA fragment by BLAST [9]. The original natural producing strain was identified as *E. coli *LM N17. The site-specific DNA endonuclease produced by the strain was named ElmI.

The PCR fragment inserted in the pElmI plasmid was sequenced. The nucleotide sequence of the fragment, 432 bp in length, was deposited in the GenBank with accession number LN869919. The sequence begins with a ATG start codon and ends with a TAA stop codon (the hypothetical reading frame has no other stop codons), and, therefore, it can be considered as an hypothetical reading frame of DNA endonuclease ElmI, and the gene encoding this protein, as *elmI*.

A comparative analysis of the sequenced fragment of the gene shows that *elmI *has essentially the same sequence as the genes encoding polypeptides of the closest homologues: *E. coli *strains BL21 (DE3) (ACT43858) and C41 (DE3) (AKN48098). The only identified substitution was the presence of cytosine at position 131 of the *elmI *gene instead of thymine in the homologous genes (*Figure 2*).

Therefore, the derived amino acid sequence of ElmI endonuclease differs from the closest homologues from the *E. coli *strains BL21 (DE3) and C41 (DE3) by one amino acid residue: ElmI has serine at position 44, whereas the closest homologues (*E. coli *BL21 (DE3) and C41 (DE3)) have leucine at the same position (*Figure 1*). At the same time, the amino acid sequence similarity between ElmI and BisI is ~50%, and the number of identical amino acids is 112%. Therefore, the cloned DNA fragment that was identified by PCR screening and represented by a putative gene of methyl-directed ElmI DNA endonuclease is highly homologous to portions of genomic DNA from well-known *E. coli* strains.

**Determination of the new ElmI MDendonuclease specificity**

In contrast to the parental strain, the lysate of *E. coli* ER2267 clones carrying pElmI plasmid exhibited endonuclease activity and one of the *E. coli *pElmI clones was chosen for production of biomass and isolation of the enzyme.

A total of 8 g of *E. coli *pElmI biomass were produced as described in the Materials and Methods section. Chromatographic purification of the biomass resulted in 3 ml of the ElmI enzyme preparation with a concentration of 4 u.a./μl.

Various substrate DNAs were digested in pre-established optimum conditions (37°C, SE reaction buffer “W”, 20 μl of the reaction mixture containing 0.5 **μ**g of substrate DNA, 2h) in order to determine the sitespecificity of ElmI. DNA was cleaved by the BisI control enzyme under the same conditions, but using the SE reaction buffer “Y.”

DNA of plasmids carrying genes of different DNA methyltransferases was used as substrates to determine the specificity of the ElmI enzyme. The activity of these genes in *E. coli *strains, from which the plasmids were isolated, resulted in modification of DNA substrates by the corresponding DNA methyltransferases, and, therefore, they had distinctive patterns of methylation.

The following plasmids were used as methylated DNA substrates:

1) pMHpaII plasmid carrying the gene encoding DNA methyltransferase HpaII, which methylates the first cytosine residues in all 5’-CCGG-3’ sequences in both strands of DNA [10];

2) pMHaeIII plasmid carrying the gene encoding DNA methyltransferase HaeIII, which methylates the first cytosine residues in all 5’-GGCC-3’ sequences in both strands of DNA [10];

3) pHspAI2 plasmid carrying the gene encoding DNA methyltransferase HspAI, which methylates the first cytosine residues in all 5’-GCGC-3’ sequences in both strands of DNA [12]. This plasmid contains an additional hypermethylated site:

5’-G(5mC)G(5mC)G(5mC)GC-3’

3’-CG(5mC)G(5mC)G(5mC)G-5’;

4) pHspAI4 plasmid also carrying the gene encoding DNA methyltransferase HspAI, which methylates the first cytosine residues in all 5’-GCGC-3’ sequences in both strands of DNA [12]. This plasmid contains an additional hypermethylated site:

5’-G(5mC)G C AG(5mC)G C-3’

3’-C G(5mC)GTC G(5mC)G-5’;

5) pFsp4HI3 plasmid carrying the gene encoding DNA methyltransferase Fsp4HI, which methylates the first cytosine residues in all 5’-GCNGC-3’ sequences [4]. This plasmid contains a hypermethylated site:

5’-G(5mC)C G(5mC)G G(5mC)A G C-3’

3’-C G G(5mC) G C(5mC)G T(5mC)G-5’.

All plasmids were pre-linearized by DriI restriction endonuclease at the 5’-GACNNNNNGTC-3’ site (unique to each plasmid).

**Fig. 3:**
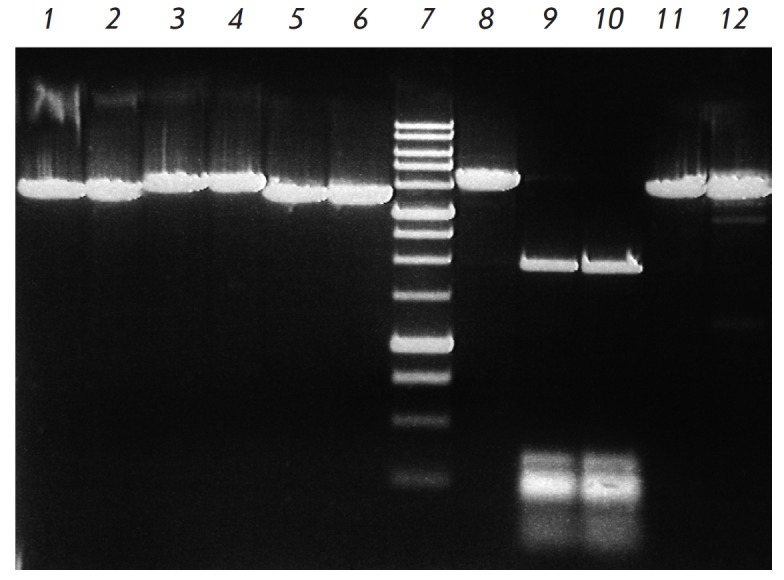
ElmI site-specificity analysis with various C5-methylated DNA substrates. Electrophoresis in 1% agarose gel. Lanes: 1 – pMHpaII/DriI; 2 – pMHpaII/DriI + ElmI; 3 – pHspAI2/DriI; 4 – HspAI2/DriI + ElmI; 5 – pMHaeIII/ DriI; 6 – pMHaeIII/DriI + ElmI; 7 – DNA molecular weight marker 1 kb; 8 – pFsp4HI3/DriI; 9 – pFsp4HI3/DriI + ElmI; 10 – pFsp4HI3/DriI + BisI. 11 – pHspAI4; 12 – pHspAI4 + ElmI.

The results of the site-specificity analysis of the DNA substrates are shown in *Figure 3*.

As can be seen from *Figure 3*, ElmI does not cleave sequences methylated by DNA methyltransferases HpaII (lane *2*), HspAI2 (lane *4*), and HaeIII (lane *6*), and, therefore, ElmI does not recognize the 5’-C(5mC) GG-3’/3’-GG(5mC)C-5’, 5’-G(5mC)GC-3’/3’-CG(5mC) G-5’, and 5’-GG(5mC)C-3’/3’-C(5mC)GG-5’ sequences.

*Figure 3* also shows that ElmI cleaves pFsp4HI3/ DriI DNA (lane *9*), producing DNA fragments of the same length as those from the treatment of the same substrate with BisI (lane *10*). Since all first cytosines residues in the 5’-GCNGC-3’ sequences of this plasmid are C5-methylated [4], and they are all digested by BisI MD-endonuclease, this suggests that ElmI recognizes and cleaves the same methylated sites. It is also important to point out that ElmI is substantially less efficient in cleaving pHspAI4 plasmid, forming two weak fragments (lane *12*). This is attributed to the fact that in contrast to pFsp4HI3 plasmid, the hypermethylated portion of pHspAI4 plasmid (see above) includes the unique 5’-GCNGC-3’sequence in which the external (second), rather than the internal (first), cytosines in both strands are methylated.

The data obtained indicate that ElmI recognizes and cleaves the methylated 5’-GCNGC-3’ sequence containing two 5-methylcytosine residues and is an order of magnitude more efficient if two internal, rather than external, cytosine residues in both strands are methylated.

**Fig. 4:**
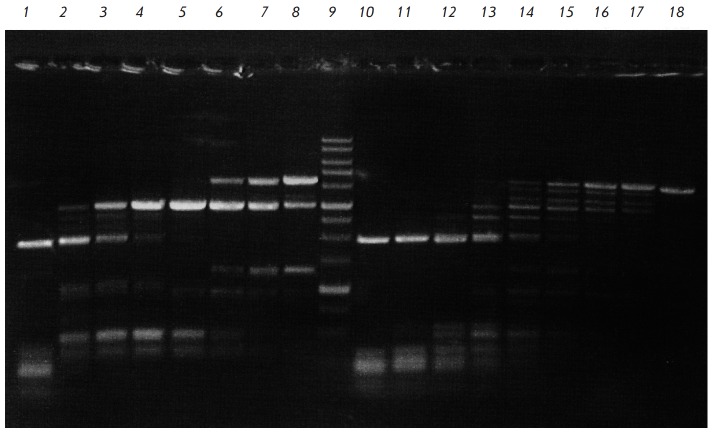
Incomplete cleavage of pFsp4HI3/DriI showing higher ElmI activity in 5’-GCNGC-3’ sequences with three or four 5-methylcytosines, compared with 5’-GCNGC-3 sequences with only two 5-methylcytosines that BisI cuts preferably. Electrophoresis in 1% agarose gel. Lanes: 1-8, pFsp4HI3/DriI, treated with serial 2-fold dilutions of ElmI (initial amount, added to the initial reaction mixture is 1 u.a.); 9, DNA molecular weight marker 1 kb; 10-17, pFsp4HI3/DriI, treated with serial 2-fold dilutions of BisI (initial amount, added to the reaction mixture is 1 u.a.); 18, pFsp4HI3/DriI.

In order to establish how effectively ElmI cleaves a 5’-GCNGC-3’ sequence with a higher number of 5-methylcytosine residues, pFsp4HI3/DriI was treated with ElmI in an amount insufficient for complete hydrolysis and the result was compared with the result of its cleavage by BisI endonuclease (*Figure 4*).

pFsp4HI3 plasmid contains two 5’-GCNGCNGC-3’ and one 5’-GCNGCNGCNGC-3’ sequences, which include, respectively, two and three intersecting Fsp4HI methylase recognition sites. Methylation of these sequences by Fsp4HIL methylase leads to a 5’-GCNGC-3’ sequence with three 5-methylcytosines or, in the case of 5’-GCNGCNGCNGC-3’, in a central 5’-GCNGC-3’ site containing four such residues. Analysis of the nucleotide sequence of pFsp4HI3 plasmid using the Vector NTI Suite 7 software shows that the cleavage of pFsp4HI3/DriI at these sequences only, as well as at the recognition site of DriI endonuclease, which was used to linearize the plasmid, should result in fragments of ~ 3000, ~ 490 (double fragment), and ~ 340 bp in size. *Figure 4* shows that these fragments are easily visualized for 2–6 dilutions of ElmI (lanes *2–5*). Even the last enzyme dilution (lane *8*) contains a fragment ~1300 bp in length, which should form by cleavage of the unique hypermethylated 5’-GCNGCNGCNGC-3’sequence, containing, among others, the 5’-GCNGC-3’ site with four 5-methylcytosine residues.

These fragments are much less visible in the case of BisI. Even though the hydrolysis of pFsp4HI3/DriI plasmid by 2–4 dilutions is much more complete compared to ElmI, the last (eighth) dilution of BisI hardly contains any ~1,300 bp fragment (Lane *17*).

These data suggest that, in contrast to BisI, the new ElmI MD-endonuclease is an order of magnitude more efficient in cleaving the 5’-GCNGC-3’ sequence in the presence of three or four 5-methylcytosine residues than in the presence of only two methylated residues. Therefore, the original DNA is completely absent after digestion with 1/16 u.a. of ElmI (lane *5*) due to a more efficient cleavage of 5’-GCNGC-3’ with three or four 5-methylcytosine residues. In contrast, BisI cleaves such hypermethylated variants less efficiently: therefore, the original DNA fragment remains visible if 1/16 u.a. is used (lane *14*).

**Determination of the position of the hydrolizable linkage in the ElmI recognition site**

The position of the hydrolizable linkage was determined by comparing the lengths of the fragments generated during the cleavage of the oligonucleotide D1/ D2 duplex, formed from oligonucleotides D1 and D2, using ElmI, PkrI, and GluI MD-endonucleases (the latter also recognizes the 5’-GCNGC-3’ methylated sequence [4] and cleaves it similarly to BisI before the central nucleotide. The putative sequence recognized by ElmI is underlined):

D1: 5’-GAGTTTAG(5mC)GG(m5C)TATCGATCC-3’

D2: 5’-GGATCGATAG(5mC)CG(m5)CTAAACTC- 3’.

**Fig. 5:**
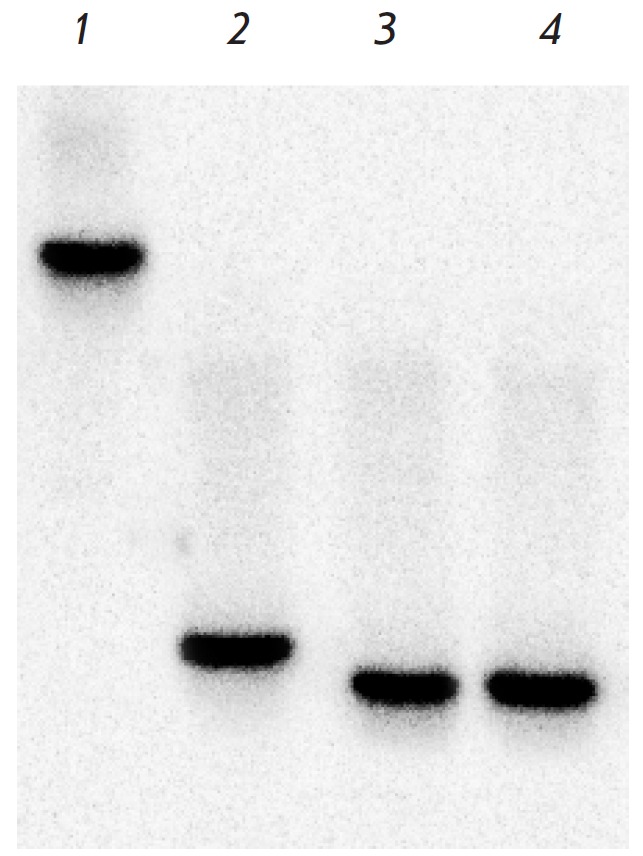
Cleavage position determination for ElmI on the oligonucleotide duplexes D1/ D2. The symbol “*” denotes the labeled chain. Electrophoresis in 20% polyacrylamide gel with 7M urea. Lanes: 1, D1*/D2; 2, D1*/D2 + PkrI; 3, D1*/D2 + ElmI; 4, D1*/D2 + GluI.

*Figure 5* shows a autoradiograph of the electropherograms of the cleavage products of the radiolabelled D1*/D2 duplex in 20% polyacrylamide gel with 7M urea.

As can be seen from *Figure 5*, the fragments derived from the hydrolysis of the D1*/D2 duplex with PkrI and ElmI (lanes *2 *and *3*, respectively) have different electrophoretic mobilities, indicating that these enzymes have different positions of hydrolizable linkage relative to the recognition site. At the same time, the electrophoretic mobilities of DNA fragments produced by ElmI and GluI are identical (lanes *3 *and *4*, respectively). Therefore, ElmI and GluI have the same position of hydrolizable linkage relative to the recognized 5’-GCNGC-3’ sequence. Since GluI cleaves the 5’-GC^NGC-3’ sequence before the central nucleotide “N” [4], ElmI also cleaves it before the central nucleotide.

## CONCLUSIONS

Thus, the first identified recombinant enterobacterial MD-endonuclease ElmI recognizes the 5’-GC^NGC-3’ nucleotide sequence and cleaves both strands of DNA before the central nucleotide “N,” producing 5’-overhanging single-nucleotide ends.

Our results indicate that enterobacterial genomes contain genes for MD-endonucleases whose amino acid sequences have only moderate homology to BisI and that only half of the amino acid residues may be regarded as similar in physical and chemical properties. Nevertheless, despite the only moderate homology of the primary structure BisI and ElmI have similar recognition sites and positions of hydrolizable linkages.

The use of the Esp-1 and Esp-2 primers and a laboratory* E. coli *BL21 (DE3) (ACT43858) strain results in amplification of an ~ 430 bp DNA fragment which is highly homologous to the *elmI *gene. According to Gen- Bank, this fragment represents a reading frame that encodes a polypeptide of unknown function: Enterobact1 - WP_001276099.1 hypothetical protein Enterobact1 – WP_001276099.1 hypothetical protein [*Escherichia coli*] >ref|YP_003035796.1| hypothetical protein ECBD_1551 [Escherichia coli ‘BL21]. However, according to our data, this reading frame is a gene encoding a methyl-directed DNA endonuclease. We have denoted the gene corresponding to this frame as *ecoBLI*, and the encoded protein as EcoBLI. Its properties will be discussed in a separate publication.

The site-specific ElmI endonuclease can be used in epigenetic studies, molecular biology, and genetic engineering for site-specific cleavage of methylated DNA: e.g., for the analysis of genomic DNA methylation in plants [13], where CNG-methylation is considered to be epigeneticaly important.

## Abbreviations

u.a.: unite activity of enzyme
MD-endonuclease: methyl-directed site specific endonuclease

## References

[1] Chernukhin V.A., Tomilova Yu.E., Chmuzh E.V., Sokolova O.O., Dedkov V.S., Degtyarev S.Kh. (2007). Bulletin of biotechnology and physico-chemical biology named by Yu.A.Ovchinnikov (Moscow). 2007. (In Russian).

[2] Chernukhin V.A., Nayakshina T.N., Gonchar D.A., Tomilova Ju.E., Tarasova M.V., Dedkov V.S., Mikhnenkova N.A., Degtyarev S.Kh. (2011). Bulletin of biotechnology and physico- chemical biology named by Yu.A.Ovchinnikov (Moscow). 2011. (In Russian).

[3] Chmuzh E.V., Kashirina J.G., Tomilova J.E., Mezentseva N.V., Dedkov V.S., Gonchar D.A., Abdurashitov M.A., Degtyarev S.Kh. (2005). Biotechnology (Moscow)..

[4] Chernukhin V.A., Chmuzh E.V., Tomilova Yu.E., Nayakshina T.N., Gonchar D.A., Dedkov V.S., Degtyarev S.Kh. (2007). Bulletin of biotechnology and physico-chemical biology named by Yu.A.Ovchinnikov (Moscow). 2007. . (In Russian).

[5] Gerhard P. (1981). Manual of methods for general bacteriology.. Manual of methods for general bacteriology. Washington, D.C.: American Society for Microbiology, 1981. 524 pages..

[6] Chambers S.P., Prior S.E., Barstow D.A., Minton N.P. (1988). Gene..

[7] Xu S.Y., Boitano M., Clark T.A., Vincze T., Fomenkov A., Kumar S., Too P.H.M., Gonchar D., Degtyarev S.Kh., Roberts R.J. (2015). Genome A..

[8] Holt J.G. (1993). Bergey’s manual of determinative bacteriology. Edited by Holt J.G.et al. 9th ed. Wiiliams and Williams, Baltimore, 1993. 787 pages..

[9] Madden T.L., Tatusov R.L., Zhang J. (1996). Meth. Enzymol..

[10] Chernukhin V.A., Kileva E.V., Tomilova Yu.E., Boltengagen A.A., Dedkov V.S., Mikhnenkova N.A., Gonchar D.A., Golikova L.N., Degtyarev S.Kh. (2011). Bulletin of biotechnology and physico-chemical biology named by Yu.A.Ovchinnikov (Moscow). 2011. (In Russian).

[11] Chernukhin V.A., Belichenko O.A., Tarasova G.V., Gonchar D.A., Akishev A.G., Dedkov V.S., Mikhnenkova N.A., Degtyarev S.Kh. (2009). Patent RU 2399663, Russia, C12N1/21, C12R1/06, 2009..

[12] Chernukhin V.A., Gonchar D.A., Kileva E.V., Sokolova. V.A., Golikova L.N., Dedkov V.S., Mikhnenkova N.A., Degtyarev S.Kh. (2012). Bulletin of biotechnology and physico- chemical biology named by Yu.A.Ovchinnikov (Moscow). 2012. (In Russian)..

[13] Vanyushin B.F. (2006). Curr. Top. Microbiol. Immunol..

